# Structural and biochemical analyses of the flagellar expression regulator DegU from *Listeria monocytogenes*

**DOI:** 10.1038/s41598-022-14459-5

**Published:** 2022-07-07

**Authors:** Han Byeol Oh, Su-jin Lee, Sung-il Yoon

**Affiliations:** Division of Biomedical Convergence, College of Biomedical Science, Kangwon National University, 1 Kangwondaehak-gil, Biomedical Science Building A-204, Chuncheon, 24341 Republic of Korea

**Keywords:** Biochemistry, Structural biology

## Abstract

*Listeria monocytogenes* is a pathogenic bacterium that produces flagella, the locomotory organelles, in a temperature-dependent manner. At 37 °C inside humans, *L. monocytogenes* employs MogR to repress the expression of flagellar proteins, thereby preventing the production of flagella. However, in the low-temperature environment outside of the host, the antirepressor GmaR inactivates MogR, allowing flagellar formation. Additionally, DegU is necessary for flagellar expression at low temperatures. DegU transcriptionally activates the expression of GmaR and flagellar proteins by binding the operator DNA in the *fliN-gmaR* promoter as a response regulator of a two-component regulatory system. To determine the DegU-mediated regulation mechanism, we performed structural and biochemical analyses on the recognition of operator DNA by DegU. The DegU-DNA interaction is primarily mediated by a C-terminal DNA-binding domain (DBD) and can be fortified by an N-terminal receiver domain (RD). The DegU DBD adopts a tetrahelical helix-turn-helix structure and assembles into a dimer. The DegU DBD dimer recognizes the operator DNA using a positive patch. Unexpectedly, unlike typical response regulators, DegU interacts with operator DNA in both unphosphorylated and phosphorylated states with similar binding affinities. Therefore, we conclude that DegU is a noncanonical response regulator that is constitutively active irrespective of phosphorylation.

## Introduction

*Listeria monocytogenes* is a gram-positive, nonspore-forming bacterium that is ubiquitously found in water, soil, plant vegetation, and animal feces and grows in a wide range of temperatures and even in low-pH and high-salt environments^1–3^. This robust bacterium contaminates most foods, including dairy products and meats, and can cause food-borne gastroenteritis or more severe diseases, such as sepsis, meningitis, and encephalitis, in humans^4–6^. *L. monocytogenes* is motile both in the environment and hosts using different locomotion modes^7,8^. At temperatures below 30 °C, *L. monocytogenes* generates flagella and moves by rotating them. However, at 37 °C in human hosts, *L. monocytogenes* stops flagellar expression and obtains locomotive force by polymerizing host actin proteins.

In *L. monocytogenes*, flagellar expression is controlled via three regulatory proteins, MogR, GmaR, and DegU^9–14^. MogR functions as a negative transcriptional regulator of all flagellar genes, and its repression activity is especially important for inhibiting flagellar production at 37 °C^12,13,15^. At or below 30 °C, GmaR binds and inactivates MogR, relieving the MogR-mediated repression of flagellar transcription^10,12^. DegU is also required to derepress flagellar transcription because DegU transcriptionally promotes GmaR expression by recognizing the operator site in the *fliN-gmaR* promoter^12,14^. Moreover, DegU directly enhances the transcription of several flagellar genes. Thus, DegU is considered a positive regulator of motility.

A two-component regulatory system (TCS) is generally used by bacteria to detect and respond to changes in the environment and cell^16,17^. The TCS typically consists of a histidine kinase and a response regulator. The histidine kinase functions as a sensor and undergoes autophosphorylation at a conserved histidine residue in response to a signal. Subsequently, the histidine kinase phosphorylates the response regulator by transferring its phosphate group to a conserved aspartate residue in the response regulator. Upon phosphorylation, the response regulator generally changes its binding affinity for the cognate operator DNA and controls genetic transcription. Interestingly, *L. monocytogenes* DegU (lmDegU) is an orphan response regulator. *L. monocytogenes* lacks the gene for the histidine kinase that phosphorylates DegU although the DegU-activating histidine kinase (DegS) has been identified in other gram-positive bacteria, such as *Bacillus subtilis*^14,18–20^.

Most response regulators are inactive in an unphosphorylated state and become activated upon phosphorylation. However, lmDegU upregulates the expression of motility genes even in an unphosphorylated state, given that the unphosphorylated lmDegU mutant still functions as a positive regulator of motility genes^9,12,21^. It is unclear how lmDegU can activate transcription even in an unphosphorylated state. Based on our structural and biochemical analyses of lmDegU using its diverse constructs and mutants, we provide the molecular mechanism in which lmDegU recognizes its operator DNA in the *fliN-gmaR* promoter and coordinates its DNA-binding activity in a domain-dependent manner. Furthermore, we demonstrate that lmDegU is a unique response regulator that exhibits significant operator DNA-binding affinity in an unphosphorylated state and does not modulate the affinity for the operator DNA in response to phosphorylation.

## Results

### Overall structure of the DNA-binding domain of lmDegU

**Figure 1:**
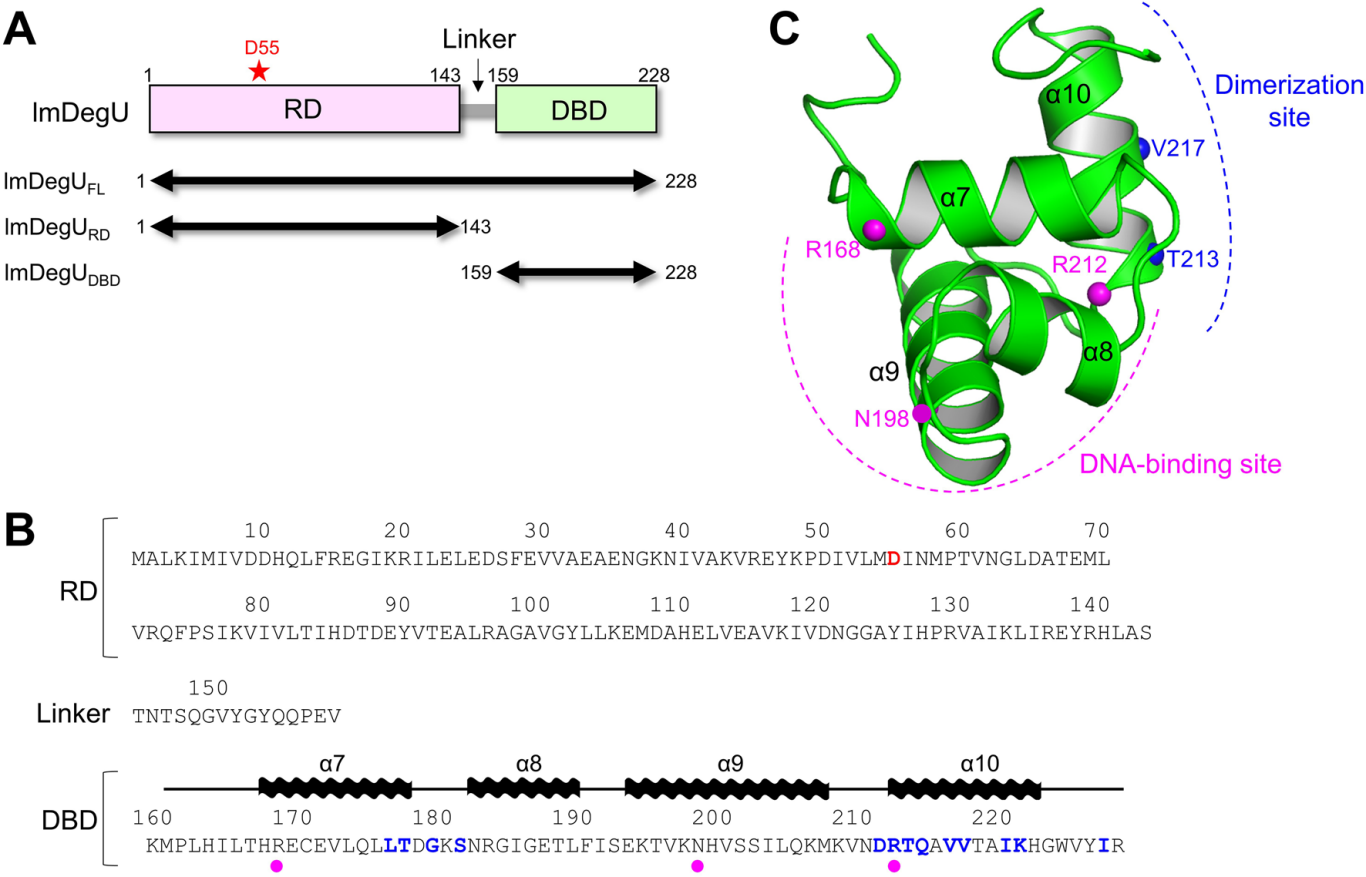
Domain organization of lmDegU and overall structure of lmDegU_DBD_. (**A**) Domain organization and expression constructs of lmDegU. The phosphorylation site of lmDegU (D55 residue) is indicated by a red star. (**B**) Amino acid sequence of lmDegU. The α-helices of the lmDegU_DBD_ structure are represented by waves above the lmDegU sequence, and the remaining region defined in the lmDegU_DBD_ structure is shown as lines. The phosphorylation site (D55 residue) and dimerization interface residues of lmDegU are colored red and blue, respectively. The three lmDegU residues (R168, N198, and R212), which were mutated to confirm their critical roles in dsDNA binding, are indicated by magenta circles. (**C**) Overall structure of a lmDegU_DBD_ monomer (chain A). The lmDegU_DBD_ structure is shown as green ribbons. The T213 and V217 residues, which were mutated to confirm the dimerization interface of lmDegU_DBD_, are shown as blue spheres. The R168, N198, and R212 residues of lmDegU, which were mutated to confirm their critical roles in dsDNA binding, are indicated by magenta spheres.

lmDegU contains an N-terminal receiver domain (RD; residues 1–143) and a C-terminal DNA-binding domain (DBD; residues 159–228), which are connected by a 15-residue linker (residues 144–158) (Fig. 1A,B and Supplementary Fig. S1). For a structural study of lmDegU to investigate operator DNA recognition by lmDegU, we expressed and purified the full-length, RD, and DBD proteins of lmDegU (lmDegU_FL_, lmDegU_RD_, and lmDegU_DBD_, respectively). The lmDegU_DBD_ protein yielded crystals that could be diffracted. The crystal structure of lmDegU_DBD_ was determined by molecular replacement and was refined to an R_free_ value of 25.3% for the X-ray diffraction data with a resolution of up to 2.39 Å (Fig. 1C, Supplementary Fig. S2, and Supplementary Table S1). The asymmetric unit of the lmDegU_DBD_ crystal contains two lmDegU_DBD_ chains (chains A and B), which have essentially identical structures with a root-mean-square deviation value of 0.50 Å (Supplementary Fig. S3).

lmDegU_DBD_ adopts a one-domain structure with a tetrahelical helix-turn-helix (HTH) motif as observed for a LuxR-type DNA-binding HTH domain that belongs to the GerE (PF00196) family in the Pfam database (Fig. 1C)^22^. The lmDegU_DBD_ structure consists of four α-helices (α7, α8, α9, and α10), and the two adjacent helices are linked by a 3–4 residue loop. The four α-helices of lmDegU_DBD_ are tethered together through interhelix hydrophobic interactions and assemble into a short rod-shaped structure. In the lmDegU_DBD_ structure, the α9 helix is the most elongated helix with 15 residues and forms a base frame that supports the other three shorter α-helices. The α9 helix is defined as a recognition helix, which has been shown to be required for dsDNA binding in GerE family members^23^.

### lmDegU_DBD_ dimerization

**Figure 2:**
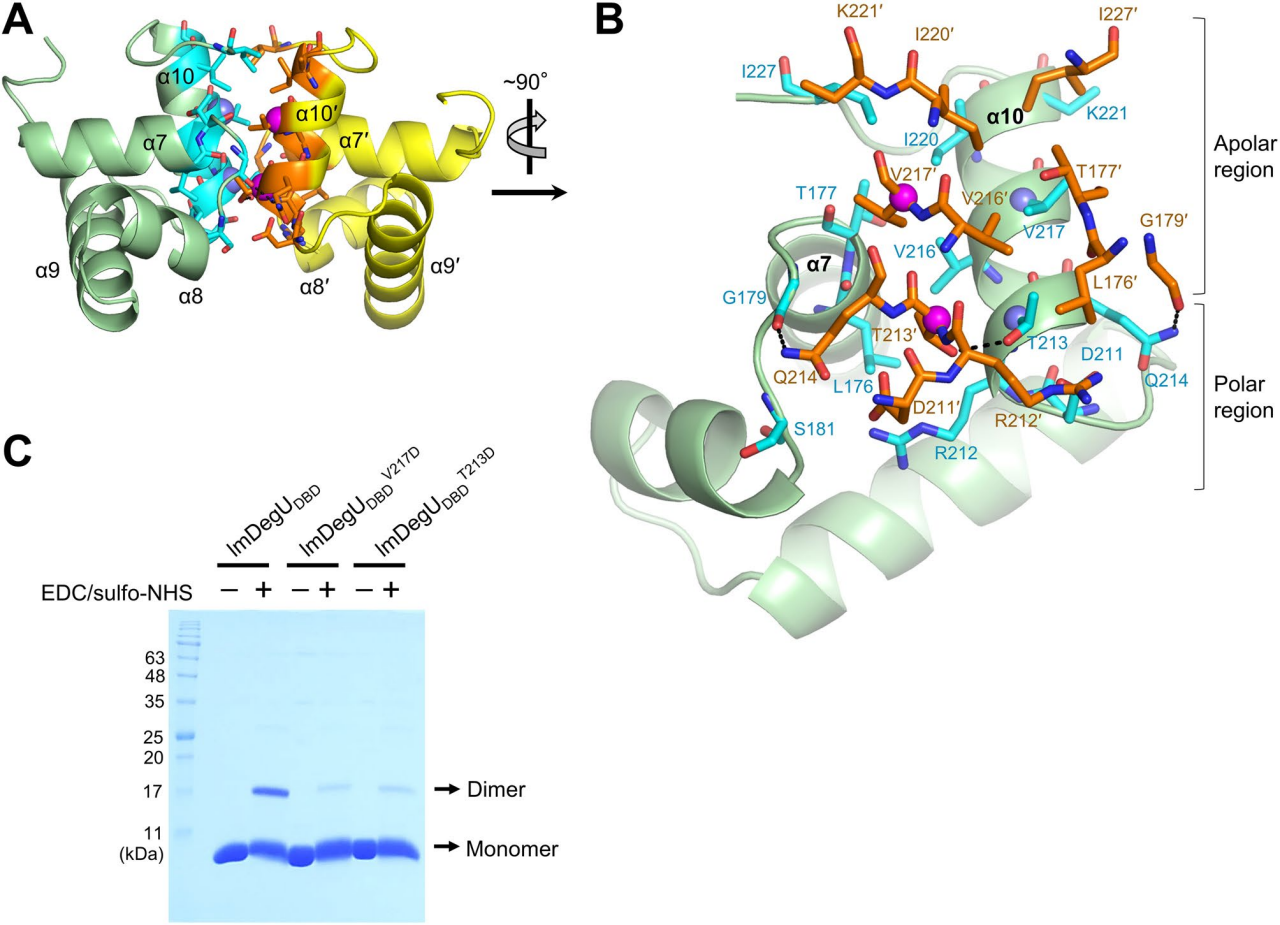
lmDegU_DBD_ dimerization. (**A**) Dimeric structure of lmDegU_DBD_. The dimerization interface residues of lmDegU_DBD_ are shown as cyan and orange sticks in the lmDegU_DBD_ dimer structure (green and yellow ribbons). The T213 and V217 residues, which were mutated to confirm the dimerization interface of lmDegU_DBD_, are highlighted by light blue or magenta spheres. (**B**) lmDegU_DBD_ residues in the dimerization interface. The dimerization interface residues from lmDegU_DBD_ chains A and B are shown as cyan and orange sticks, respectively. lmDegU_DBD_ chain A is depicted as green ribbons. Intermolecular hydrogen bonds are represented by dashed lines. The T213 and V217 residues, which were mutated to confirm the dimerization interface of lmDegU_DBD_, are highlighted by light blue or magenta spheres. (**C**) lmDegU_DBD_ dimerization and its disruption by the mutation of dimerization interface residues (T213D and V217D). lmDegU_DBD_ or its mutant was crosslinked using EDC and sulfo-NHS and analyzed by SDS-PAGE. Protein bands were identified by Coomassie brilliant blue staining. The gel image is representative of three independent experiments that yielded similar results. The full-length gel is shown in Supplementary Fig. S10. The V217D and T213D mutations do not seem to significantly modulate the folding of the lmDegU_DBD_ protein, as lmDegU_DBD_^V217D^ and lmDegU_DBD_^T213D^ displayed CD spectra similar to that of lmDegU_DBD_ (Supplementary Fig. S6). Moreover, lmDegU_DBD_^V217D^ and lmDegU_DBD_^T213D^ were eluted as single peaks in gel-filtration chromatography in elution volumes similar to that of lmDegU_DBD_ (Supplementary Fig. S5).

Two polypeptide chains in the asymmetric unit of the lmDegU_DBD_ crystal form a dimer (Fig. 2A). The dimerization interface of lmDegU_DBD_ (buried surface area, ~ 500 Å^2^) is mainly located at the α10 helix with additional contributions from the C-terminal region of the α7 helix and the α7-α8 and α9-α10 loops (Figs. 1B and 2A). The α10 helix is parallelly aligned with its equivalent helix from the dimerization partner (α10′; the primer denotes the second lmDegU_DBD_ chain in the dimer structure) and is responsible for ~ 64% of the dimerization interface. The dimerization interface of lmDegU_DBD_ is constituted by apolar and polar residues, which are primarily separated into the upper and lower regions of the dimerization interface, respectively (Fig. 2B). Noticeably, the G179 residue in the dimerization interface is most conserved in GerE family sequences and can be defined as a canonical residue of the GerE family (Supplementary Fig. S4). The G179 residue at the α7-α8 loop also functions as a structural residue that is required for the α7-α8 loop to form a sharp turn.

The dimer formation of lmDegU_DBD_ was verified in solution by a chemical crosslinking experiment (Fig. 2C). SDS-PAGE analysis of the crosslinked and non-crosslinked lmDegU_DBD_ proteins indicated that the molecular size of lmDegU_DBD_ shifted to that of a dimer in the presence of crosslinking reagents. However, the dimerization affinity of lmDegU_DBD_ seems to be low, given that the dimeric form of lmDegU_DBD_ was not detected in gel-filtration chromatography, which cannot identify low-affinity interactions (Supplementary Fig. S5). Consistently, the lmDegU_DBD_ dimer has a relatively small buried surface area of ~ 500 Å^2^.

To confirm the dimerization interface found in the lmDegU_DBD_ crystal, the T213 and V217 residues located in the center of the lmDegU_DBD_ dimerization interface were individually mutated to a larger negatively charged residue, aspartate (Fig. 2A,B). The mutation was expected to disrupt the apolar interaction in the dimerization interface of lmDegU_DBD_. Indeed, in the crosslinking experiment, the lmDegU_DBD_^V217D^ and lmDegU_DBD_^T213D^ mutants exhibited substantially lower dimerization efficiencies than lmDegU_DBD_ (Fig. 2C and Supplementary Fig. S6). However, when the lmDegU_DBD_ residues that are located at the periphery of the dimerization interface (R212) or outside the dimerization interface (R168 and N198) were mutated, the dimerization efficiency of lmDegU_DBD_ did not change (Fig. 1B and Supplementary Fig. S7). These results demonstrate that the central dimerization interface of lmDegU_DBD_, involving the T213 and V217 residues, plays a key role in lmDegU_DBD_ dimerization.

### DNA recognition by lmDegU using the DBD

**Figure 3:**
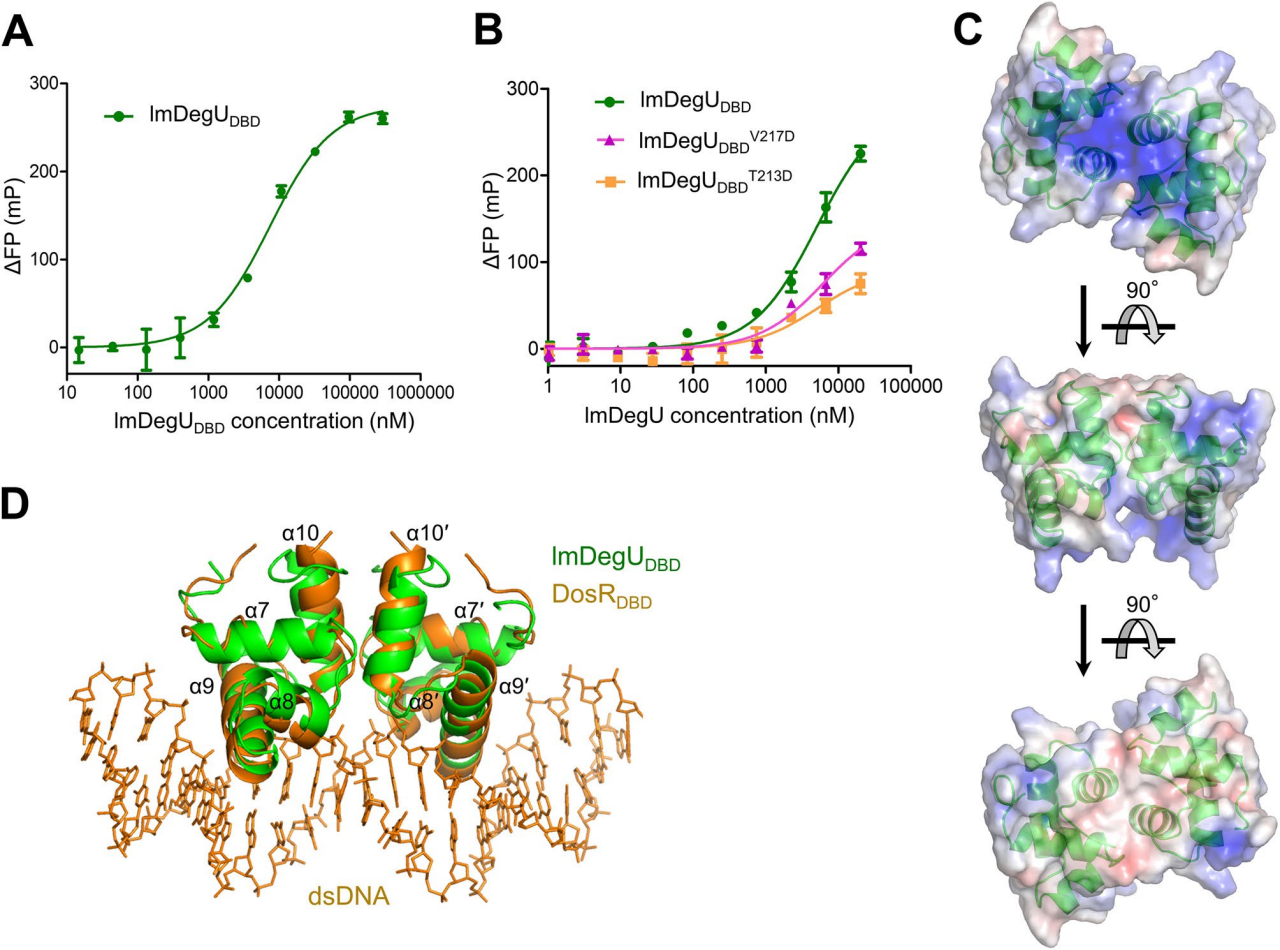
DNA binding by lmDegU_DBD_. (**A**) Operator dsDNA-binding affinity of lmDegU_DBD_ based on the FP assay. The data (means ± S.D.) are representative of four independent experiments that yielded similar results. (**B**) DNA-binding levels of lmDegU_DBD_ and the dimerization-deficient mutants (lmDegU_DBD_^T213D^ and lmDegU_DBD_^V217D^) based on the FP assay. The data (means ± S.D.) are representative of three independent experiments that yielded similar results. (**C**) Surface electrostatic potentials of lmDegU_DBD_. The lmDegU_DBD_ dimer structure is shown as semi-transparent electrostatic potential surfaces (positive, blue; neutral, white; negative, red) with ribbons (green). The orientation of lmDegU_DBD_ in the middle panel is identical to that in Fig. 2A. (**D**) Overlay of the lmDegU_DBD_ dimer structure (green ribbons) on the DosR_DBD_-dsDNA complex structure (DosR_DBD_, orange ribbons; dsDNA, orange lines; PDB ID 1ZLK)^24^. The orientation of the lmDegU_DBD_ dimer in the figure is identical to that in the middle panel in (**C**).

lmDegU was shown to interact with the operator site in the *fliN-gmaR* promoter^12^. To examine whether lmDegU employs its DBD to interact with the operator dsDNA, a fluorescence polarization (FP) assay was performed using the fluorescein-labeled operator dsDNA that contains a palindromic sequence from the *fliN-gmaR* promoter (Fig. 3A). The lmDegU_DBD_ protein interacted with dsDNA but with a relatively low binding affinity (dissociation constant K_d_, 6.7 ± 2.3 μM). Given that lmDegU_DBD_ binds the palindromic DNA sequence, it is highly likely that lmDegU_DBD_ recognizes dsDNA as a dimeric form. To examine whether lmDegU_DBD_ employs its dimeric structure for the dsDNA interaction, the lmDegU_DBD_^T213D^ and lmDegU_DBD_^V217D^ mutants that are deficient in dimerization were subjected to an FP assay (Fig. 3B). Both the lmDegU_DBD_^T213D^ and lmDegU_DBD_^V217D^ mutants displayed lower dsDNA-binding activity than the dimerization-competent lmDegU_DBD_ protein, indicating that the dimeric assembly and organization observed in the crystal structure of lmDegU_DBD_ are utilized to achieve the optimal interaction of lmDegU with dsDNA.

**Figure 4:**
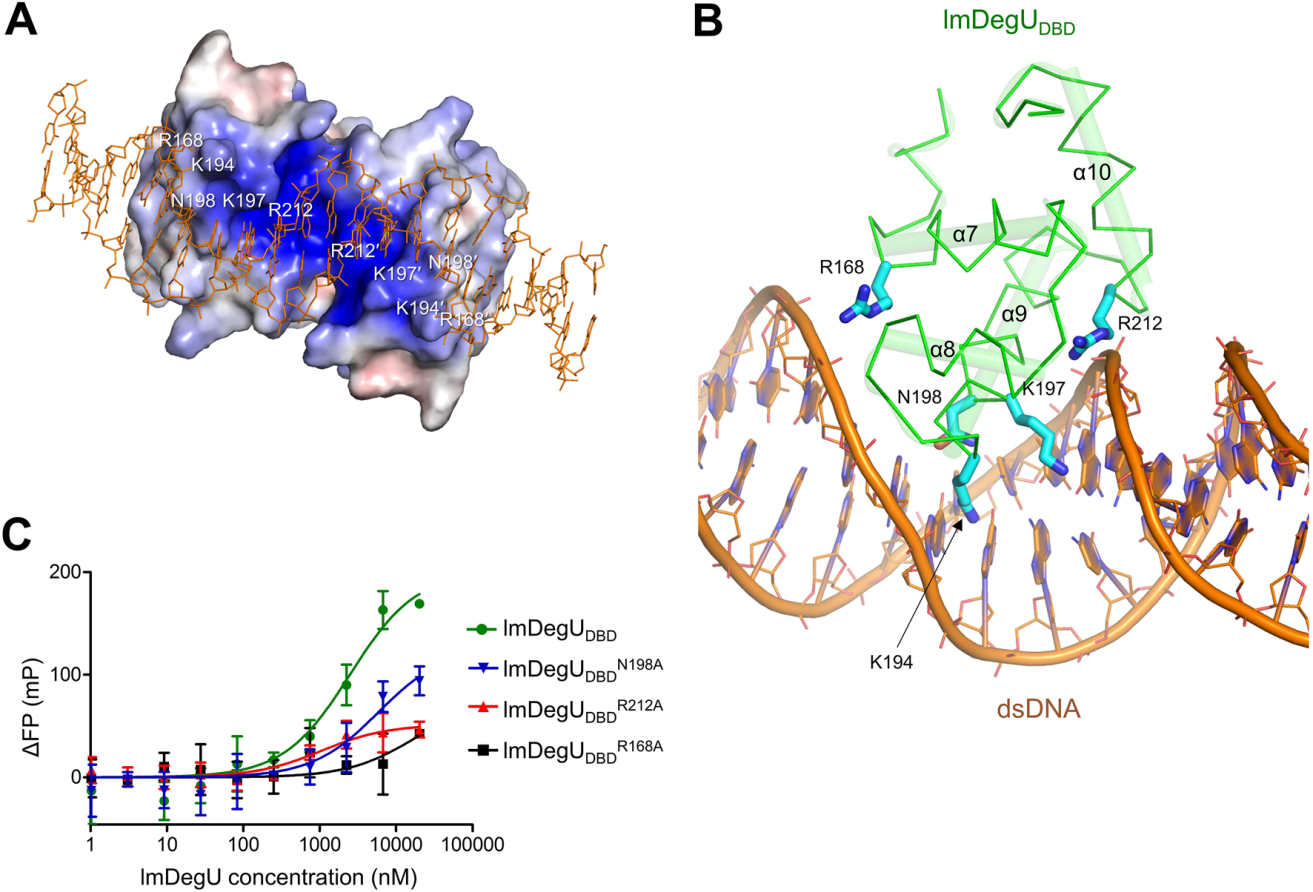
dsDNA-binding residues of lmDegU_DBD_. (**A**) Putative dsDNA-binding residues of lmDegU_DBD_ (white labels) in the model of a complex between the lmDegU_DBD_ dimer (electrostatic potential surfaces) and dsDNA (orange lines). In the model, dsDNA resides on the positive electrostatic potential surface of the lmDegU_DBD_ dimer. (**B**) Interactions of the lmDegU R168, K194, K197, N198, and R212 residues (cyan sticks) with dsDNA in the model of a complex between the lmDegU_DBD_ monomer (green Cα traces with transparent cylindrical helices) and dsDNA (orange lines and cartoons with filled base rings). (**C**) Mutagenesis-based verification of the dsDNA-binding surface of lmDegU_DBD_. The dsDNA-binding affinities of the lmDegU_DBD_^R168A^, lmDegU_DBD_^N198A^, and lmDegU_DBD_^R212A^ mutants were analyzed by the FP assay in comparison with that of lmDegU_DBD_. The data (means ± S.D.) are representative of five independent experiments that yielded similar results.

To be consistent with the dsDNA-binding ability of lmDegU_DBD_, the lmDegU_DBD_ dimer structure displays a continuous positive patch that is electrostatically complementary to the negatively charged dsDNA (Fig. 3C). To address the DNA-binding mode of lmDegU, we overlaid the lmDegU_DBD_ dimer structure on the DBD of the lmDegU homolog DosR (DosR_DBD_) in complex with dsDNA and generated a lmDegU_DBD_-dsDNA model by combining the lmDegU_DBD_ structure with the dsDNA structure from the DosR_DBD_-dsDNA complex (Fig. 3D)^24^. In the 2:1 lmDegU_DBD_-dsDNA model, dsDNA resides on the positive patch of the lmDegU_DBD_ dimer structure (Fig. 4A). In particular, the α9 recognition helix is inserted into the major groove of dsDNA and appears to play a critical role in dsDNA recognition (Fig. 3D). The positively charged K194 and K197 residues from the α9 helix are located in the positive patch and interact with the DNA bases in the major groove of dsDNA in the complex model (Fig. 4A,B). The neutral hydrophilic residue N198 at the periphery of the positive patch from the α9 helix also seems to function as a DNA base binder. In addition to α9-helix residues, several lmDegU residues from the α7 and α10 helices and their neighboring loops contribute to the interaction of lmDegU with the backbone of dsDNA. In the complex model, the positively charged R168 and R212 residues from the α7 and α10 helices, respectively, make contacts with the backbone of dsDNA. These five lmDegU residues (R168, K194, K197, N198, and R212) are highly conserved in lmDegU orthologs (Supplementary Fig. S1). Notably, among the putative dsDNA-binding residues, the lmDegU R168 residue is conserved as a positively charged residue (arginine or lysine) even across GerE family sequences, suggesting that the R168 residue is indispensable for sequence-independent interactions with dsDNA, such as phosphate recognition (Supplementary Fig. S4).

To confirm the dsDNA-binding residues observed in the lmDegU_DBD_-dsDNA model, the R168, N198, and R212 residues at the putative dsDNA-binding site were individually mutated to alanine in lmDegU_DBD_. Each of the three lmDegU_DBD_ mutants (lmDegU_DBD_^R168A^, lmDegU_DBD_^N198A^, and lmDegU_DBD_^R212A^) exhibited lower dsDNA-binding affinity in the FP assay than lmDegU_DBD_, indicating that the continuous positive patch of the lmDegU_DBD_ dimer covering the R168, R212, and N198 residues mediates dsDNA recognition (Fig. 4C and Supplementary Fig. S6). Noticeably, among the three mutants, the lmDegU_DBD_^R168A^ mutant displayed the lowest dsDNA binding level, highlighting the critical role of the highly conserved, positively charged R168 residue in DNA recognition.

### Contribution of the lmDegU RD to the DNA-binding capacity of lmDegU

**Figure 5:**
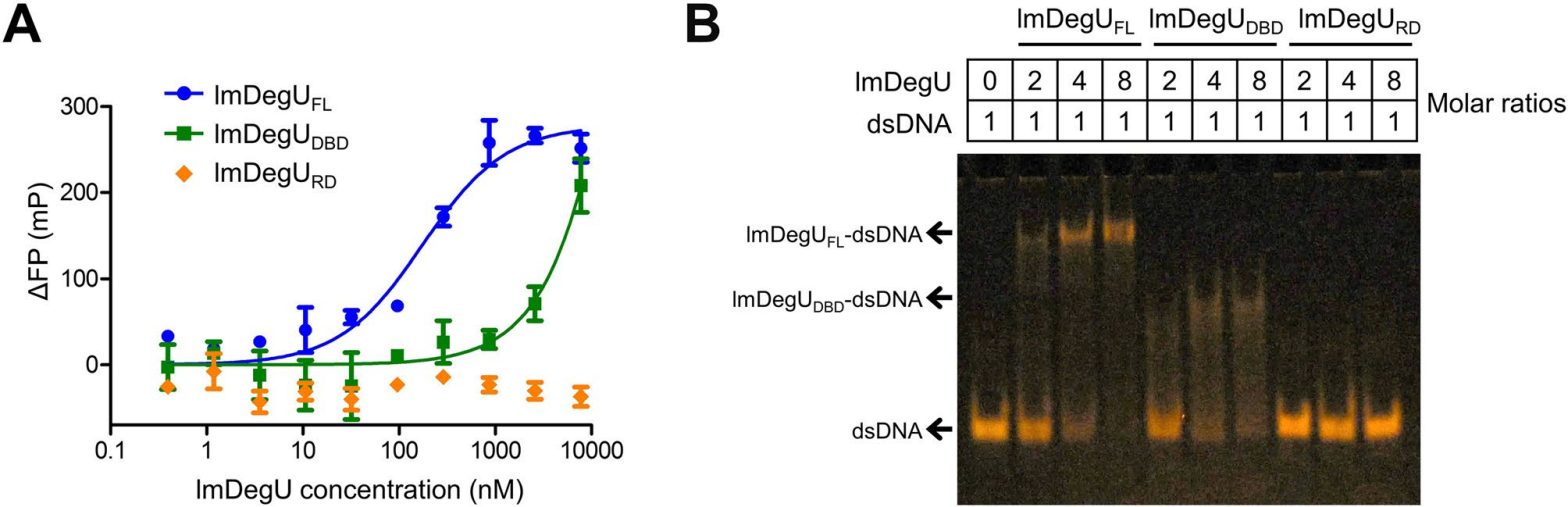
Enhancement of the DNA-binding capacity of lmDegU by the RD. (**A**) dsDNA-binding levels of lmDegU_FL_, lmDegU_DBD_, and lmDegU_RD_ based on the FP assay. The data (means ± S.D.) are representative of three independent experiments that yielded similar results. (**B**) dsDNA-binding capacities of lmDegU_FL_, lmDegU_DBD_, and lmDegU_RD_ based on the EMSA. The gel image is representative of three independent experiments that yielded similar results. The full-length gel is shown in Supplementary Fig. S11.

To determine the relative contributions of the lmDegU DBD and RD to the dsDNA interaction, an FP assay was performed using the lmDegU_DBD_ and lmDegU_RD_ proteins containing only one domain (Figs. 1A,B and 5A). The lmDegU_RD_ protein did not exhibit any detectable dsDNA binding up to a 7.8 μM concentration, whereas the lmDegU_DBD_ protein obviously interacted with dsDNA (K_d_, 6.7 ± 2.3 μM) (Figs. 3A and 5A). This observation indicates that the direct interaction of lmDegU with dsDNA is primarily mediated by the DBD. Interestingly, compared to lmDegU_DBD,_ the lmDegU_FL_ protein containing the RD and DBD more potently interacted with dsDNA (K_d_, 173 ± 26 nM) by ~ 39-fold in the FP assay, suggesting that the RD makes an indirect contribution to operator DNA binding. This dsDNA-binding pattern of lmDegU was recapitulated in an electrophoresis mobility shift assay (EMSA) (Fig. 5B). In the EMSA, lmDegU_RD_ could not shift the dsDNA band to the lmDegU_RD_-dsDNA complex band. lmDegU_DBD_ interacted with dsDNA but with a partial shift to the complex band even at an 8:1 lmDegU:dsDNA molar ratio. In contrast, a complete shift was observed for lmDegU_FL_, indicating that lmDegU_FL_ binds dsDNA with a higher affinity than lmDegU_DBD_.

### Phosphorylation-independent DNA-binding capacity of lmDegU

**Figure 6:**
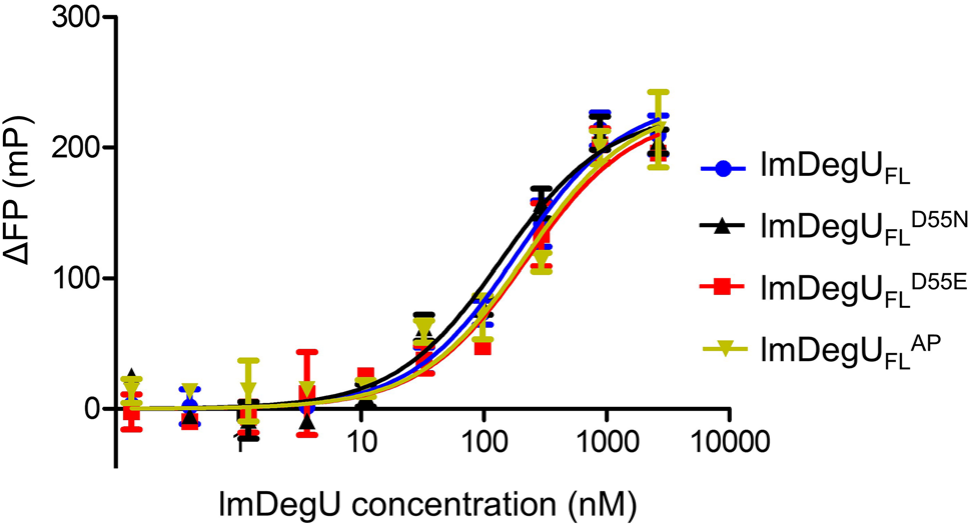
Similar dsDNA-binding levels of lmDegU observed irrespective of phosphorylation. The dsDNA-binding levels of lmDegU_FL_, phosphorylation-incompatible lmDegU_FL_^D55N^, the lmDegU_FL_^D55E^ phosphomimetic, and acetyl phosphate-phosphorylated lmDegU_FL_ (lmDegU_FL_^AP^) were determined by the FP assay. The data (means ± S.D.) are representative of five independent experiments that yielded similar results.

Most response regulators control their interactions with operator DNA and subsequent transcription in a phosphorylation-dependent manner^16,17^. In general, response regulators do not recognize operator DNA when they are not phosphorylated. Upon phosphorylation, response regulators enhance their operator DNA-binding capacity via a conformational change and homodimerization. In contrast to typical response regulators, the unphosphorylated lmDegU_FL_ protein displayed substantial binding to the operator dsDNA with a K_d_ value of 173 ± 26 nM (Figs. 5A and 6). To rule out the possibility that the lmDegU_FL_ protein used in the assay was already phosphorylated during expression or purification, a lmDegU_FL_ mutant (lmDegU_FL_^D55N^) that cannot be phosphorylated due to a phosphorylation site mutation (D55N mutation) was generated and analyzed for dsDNA binding (Fig. 1B). The lmDegU_FL_^D55N^ mutant exhibited comparable dsDNA binding to that of lmDegU_FL_ in the FP assay, confirming the ability of unphosphorylated lmDegU to recognize operator DNA (Fig. 6 and Supplementary Fig. S6).

To address any phosphorylation-mediated changes in the lmDegU-dsDNA interaction, the phosphorylated lmDegU_FL_ protein was generated using acetyl phosphate as a phosphodonor, and its dsDNA binding was analyzed by an FP assay. Acetyl phosphate has been shown to phosphorylate diverse response regulators, including lmDegU, in vitro^25–27^. Unexpectedly, the phosphorylated lmDegU_FL_ protein displayed essentially identical dsDNA binding to that of unphosphorylated lmDegU_FL_ (Fig. 6). Moreover, mutation of the aspartate residue at the phosphorylation site (D55) to glutamate to generate the phosphomimetic lmDegU_FL_ (lmDegU_FL_^D55E^) did not significantly change the dsDNA-binding affinity of lmDegU_FL_ (Fig. 6 and Supplementary Fig. S6). Considering these results, we conclude that lmDegU is a unique response regulator that interacts with the operator dsDNA even in an unphosphorylated state and does not change its dsDNA-binding ability in response to phosphorylation.

Despite the phosphorylation-independent lmDegU-dsDNA interaction, phosphorylation appears to modulate the lmDegU structure. In gel-filtration chromatography, the lmDegU_FL_^D55E^ phosphomimetic was eluted earlier than lmDegU_FL_, suggesting that phosphorylation induces a change in the conformation or size of lmDegU (Supplementary Fig. S8). Furthermore, given that lmDegU contains the key residues (D50, T83, Y102, and K105 residues) required for phosphorylation-mediated conformational rearrangement and dimerization, it is highly likely that lmDegU undergoes the structural changes that have been reported in typical response regulators.

## Discussion

lmDegU plays a key role in the transcriptional activation of GmaR protein and flagellar proteins^12^. This transcriptional regulation is mediated by the interaction of lmDegU with its operator dsDNA in the *fliN-gmaR* promoter. Our structural and biochemical studies indicate that lmDegU recognizes the operator dsDNA using the DBD in a dimeric organization. This binding is fortified by the RD. The RD-mediated increase in dsDNA-binding affinity was also reported in other NarL family members that are homologous to lmDegU. For example, full-length LiaR bound the operator dsDNA with 100-fold higher affinity than its DBD^28^.

lmDegU displayed significant dsDNA-binding capacity although it was not phosphorylated. Consistently, lmDegU was demonstrated to induce flagellar and GmaR expression even without phosphorylation^12,26^. In contrast to lmDegU, VraR and NarL, which belong to the NarL family, were shown to exist in an autoinhibited conformation via RD-mediated occlusion of the DBD dimerization site or the DNA-binding site when unphosphorylated^29,30^. Thus, unphosphorylated VraR did not exhibit any detectable dsDNA binding^29^. NarL family members use phosphorylation as a key mechanism to regulate their activities. VraR changes from an inactive conformation to an active form upon phosphorylation by releasing the DBD from the RD to interact with dsDNA^29^. As a result, phosphorylation enhanced the dsDNA-binding affinity of VraR by at least 30-fold. However, lmDegU phosphorylation did not significantly improve the DNA-binding capacity, given that the lmDegU phosphomimetic and chemically phosphorylated lmDegU displayed similar DNA-binding affinities to that of the unphosphorylated lmDegU protein. This comparative analysis indicates that lmDegU displays a unique phosphorylation-independent dsDNA-binding mode that is not observed in NarL and VraR despite 30–40% sequence identities of lmDegU shared with NarL and VraR.

The interdomain helix, α6, which is located between the RD and DBD, was proposed to be a critical player that determines VraR activity^29,31^. In unphosphorylated VraR, the α6 helix tethers the RD and DBD into the inactive conformation by bridging the two domains. Upon phosphorylation-mediated activation, the α6 helix undergoes structural rearrangements to liberate the DBD for DNA binding. Interestingly, lmDegU contains an exceptionally long interdomain region (Supplementary Fig. S9). Thus, we hypothesize that the additional interdomain region positions the DBD away from the RD without an interdomain interaction in the unphosphorylated state and contributes to the adoption of the active conformation even without phosphorylation. We could not prove this hypothesis because of a technical difficulty. Specifically, a lmDegU mutant lacking the additional loop region could not be obtained due to protein instability. Future structural studies on lmDegU in both unphosphorylated and phosphorylated states are necessary to reveal the exact mechanism in which lmDegU adopts the active state without phosphorylation.

The regulation of flagellar expression by unphosphorylated lmDegU is not specific to *L. monocytogenes*. In *B*. *subtilis*, unphosphorylated DegU positively regulates the *fla*/*che* operon and *comK* gene for flagellar formation and genetic competence, respectively^32,33^. Another common property of unphosphorylated lmDegU and *B*. *subtilis* DegU (bsDegU) is that both recognize inverted repeat sequences. However, when phosphorylated, bsDegU controls the expression of another set of ~ 170 genes, including genes involved in degradative enzyme production, potentially by recognizing direct repeat sequences^34,35^. Therefore, unlike lmDegU, bsDegU is considered a molecular switch that alternatively activates transcription of two gene sets depending on phosphorylation^36^. In addition to this regulatory distinction, *L. monocytogenes* differs from *B. subtilis* in that *L. monocytogenes* lacks the *degS* gene that is required to phosphorylate DegU. Thus, *L. monocytogenes* and *B. subtilis* seem to have undergone different evolutionary routes in the DegS-DegU system although both species belong to the same order Bacillales in the phylum Firmicutes.

lmDegU can be phosphorylated by acetyl phosphate in *L. monocytogenes*, and lmDegU phosphorylation was shown to accelerate flagellar expression^26^. However, in our binding study, phosphorylation did not enhance the dsDNA-binding affinity of lmDegU, indicating that the phosphorylation-mediated improvement in flagellar expression is not ascribed to the direct interaction of lmDegU with the operator DNA in the *fliN-gmaR* promoter. Notably, phosphorylation modulated the elution profile of lmDegU in gel-filtration chromatography, indicating that lmDegU changes its conformation or oligomeric state upon phosphorylation (Supplementary Fig. S8). These observations lead us to propose that the phosphorylation-mediated structural change in lmDegU generates a new surface that can be used to recruit an unidentified regulator and to improve motility gene expression.

## Methods

### Construction of the protein expression plasmid

The DNA fragment that encodes the lmDegU_FL_ protein (residues 1–228) was amplified by PCR from the genomic DNA of *L. monocytogenes* ATCC 15313. The PCR product was digested using the *Nde*I and *Xma*I restriction enzymes and was inserted using T4 DNA ligase into the pET49b plasmid that was modified to express the recombinant protein in fusion with a C-terminal hexahistidine (His_6_) tag. The ligation product was transformed into the *E. coli* DH5α strain. A transformant containing the lmDegU_FL_ expression plasmid was verified by DNA sequencing. The lmDegU_RD_ (residues 1–143) and lmDegU_DBD_ (residues 159–228) expression plasmids were generated by PCR using the lmDegU_FL_ expression plasmid as template DNA and by the subsequent ligation of the *Bam*HI- and *Sal*I-digested PCR product into the pET49b vector, which was modified to express recombinant protein with an N-terminal His_6_ tag and a subsequent thrombin or TEV protease cleavage site^37^. The lmDegU gene in the expression plasmid was mutated using the site-directed mutagenesis protocol (Agilent).

### Protein expression and purification

For protein overexpression, the lmDegU expression plasmid was transformed into the *E. coli* BL21 (DE3) strain. *E. coli* BL21 (DE3) cells containing the lmDegU expression plasmid were grown at 37 °C in LB broth containing 100 μM kanamycin. When the optical density of the culture at 600 nm reached 0.6, the culture was supplemented with 1 mM isopropyl β-d-1-thiogalactopyranoside for overexpression. The cells were further grown at 18 °C for 18 h. The resulting cells were lysed by sonication in a solution containing 50 mM Tris, pH 8.0, 300 mM sodium chloride, and 5 mM β-mercaptoethanol. The lmDegU protein was first purified from the cell lysate by Ni–NTA affinity chromatography through imidazole-mediated elution. The eluted lmDegU_FL_, lmDegU_RD_, and lmDegU_DBD_ proteins were dialyzed against solutions of different compositions (50 mM Tris, pH 8.0, 300 mM sodium chloride, and 5 mM β-mercaptoethanol for lmDegU_FL_; 20 mM Tris, pH 8.0, and 5 mM β-mercaptoethanol for lmDegU_RD_; 20 mM Hepes, pH 7.4, 300 mM sodium chloride, and 5 mM β-mercaptoethanol for lmDegU_DBD_). The dialyzed lmDegU_RD_ and lmDegU_DBD_ proteins were subjected to digestion by TEV protease and thrombin, respectively, to cleave the His_6_ tag. The tag-free lmDegU_RD_ and lmDegU_DBD_ proteins were further purified by gel-filtration chromatography using a Superdex 200 16/600 column (GE Healthcare) in solutions with different compositions (20 mM Tris, pH 8.0, and 150 mM sodium chloride for lmDegU_RD_; 20 mM Hepes, pH 7.4, 300 mM sodium chloride, and 5 mM β-mercaptoethanol for lmDegU_DBD_).

### Crystallization and X-ray diffraction

The purified lmDegU_DBD_ protein was concentrated to 11.6 mg/ml for crystallization. lmDegU_DBD_ crystals were obtained by performing a sitting-drop vapor-diffusion method using a 24-well Cryschem plate (Hampton Research). For crystallization, 0.5 μl of the lmDegU_DBD_ protein was mixed with 0.5 μl of a well solution containing 22% PEG 3350 and 0.1 M Tris, pH 8.0, and was equilibrated via vapor diffusion against 500 μl of the well solution at 18 °C. A lmDegU_DBD_ crystal was briefly soaked in 25% glycerol, 24% PEG 3350, and 0.1 M Tris, pH 8.0, for cryoprotection and flash-cooled at − 173 °C under a nitrogen gas stream. X-ray diffraction data from a single lmDegU_DBD_ crystal were collected at beamline 7A, Pohang Accelerator Laboratory. The X-ray diffraction data were processed and scaled using the HKL2000 program^38^. The data collection statistics are listed in Supplementary Table S1.

### Structure determination and analysis

The lmDegU_DBD_ structure was determined by molecular replacement with the Phaser program^39^. Molecular replacement was performed using the crystal structure of the DNA-binding domain of *Enterococcus faecalis* LiaR (PDB ID 4WSZ) as a search model^40^. The initial model of lmDegU_DBD_ was iteratively modified and refined using the Coot and phenix.refine programs, respectively^41,42^. TLS refinement was performed using 6 TLS groups during the refinement runs to generate the lmDegU_DBD_ structure. The final structure of lmDegU_DBD_ exhibited good geometry and stereochemistry without Ramachandran plot outliers. The lmDegU_DBD_ structure has relatively high B-factors (average B-factor, 76.6 Å), presumably due to inherent intersubunit flexibility that is caused by the low dimerization affinity. The refinement statistics are listed in Supplementary Table S1.

### Chemical crosslinking of lmDegU_DBD_

lmDegU_DBD_ dimerization was verified by crosslinking. For chemical crosslinking, 5 μg of lmDegU_DBD_ or its mutants in 5 μl of 20 mM Hepes, pH 7.4, 300 mM sodium chloride, and 5 mM β-mercaptoethanol was incubated with a 5-μl mixture of 80 mM 1-ethyl-3-(3-dimethylaminopropyl)carbodiimide hydrochloride (EDC) and 80 mM N-hydroxysulfosuccinimide (Sulfo-NHS) for 5 min at room temperature. The crosslinking reaction was stopped using 5 μl of a solution containing 500 mM Tris, pH 8.0, and 20 mM β-mercaptoethanol. The crosslinked protein was analyzed by SDS-PAGE and stained using Coomassie Brilliant Blue G-250 dye.

### FP assay

To determine the dsDNA-binding affinity of lmDegU, an FP assay was performed. For the FP assay, an operator dsDNA was generated by annealing a fluorescein-labeled 36-mer ssDNA fragment (5′-CGAGTAGGTCAAAAGGATTGGGTATGAAGAACCTTT-3′ in the *fliN-gmaR* promoter site) and its unlabeled complementary ssDNA counterpart (5′-AAAGGTTCTTCATACCCAATCCTTTTGACCTACTCG-3′)^12^. The resultant 36-bp operator dsDNA (0.3 nM) was incubated with lmDegU protein at various concentrations for 30 min at 18 °C in 20 mM Tris, pH 7.0, 50 mM sodium chloride, and 5 mM β-mercaptoethanol^12^. The fluorescence polarization of the fluorescein-labeled dsDNA in the absence and presence of lmDegU protein was measured using an Infinite F200 PRO instrument (Tecan) and analyzed with the Prism 5 software (GraphPad) using a one-site binding model to derive a K_d_ value for the lmDegU-dsDNA interaction.

### EMSA

To qualitatively analyze the lmDegU-dsDNA interaction, an EMSA was performed using the lmDegU protein and unlabeled 36-bp operator dsDNA. The lmDegU protein was incubated with the operator dsDNA at various molar ratios at 18 °C for 30 min. The protein-dsDNA mixture was electrophoresed in a polyacrylamide gel using Tris–borate-EDTA running buffer. DNA bands in the electrophoretic gel were visualized by ethidium bromide.

### Gel-filtration chromatography analysis

Gel-filtration chromatography was performed to analyze the molecular size and folding of lmDegU. The lmDegU protein in 50 mM Tris, pH 8.0 (or 20 mM Hepes, pH 7.4), 300 mM sodium chloride, and 5 mM β-mercaptoethanol was loaded onto a Superdex 200 10/300 column. Protein elution was monitored by measuring the UV absorbance at 280 nm. For comparison, a gel-filtration standard solution (Bio-Rad) was independently loaded onto the column.

### Circular dichroisms (CD) spectroscopy

To verify that mutation does not affect protein folding, CD spectra were obtained using lmDegU_FL_, lmDegU_DBD_, and their mutants (0.5 mg/ml). The purified lmDegU protein was dialyzed against a solution containing 50 mM Tris, pH 8.0, 300 mM sodium fluoride, and 5 mM β-mercaptoethanol and then subjected to CD measurement. CD spectra from 190 to 260 nm were recorded at 25 °C using a J-1500 CD spectropolarimeter (Jasco) at the Korea Basic Science Institute (Ochang, Korea), with a step resolution of 0.1 nm, a bandwidth of 1 nm, and a response time of 1 s.

### Structure deposition

The atomic coordinates and the structure factors for lmDegU_DBD_ (PDB ID 7X1K) have been deposited in the Protein Data Bank (http://www.rcsb.org).

## Supplementary Information

Supplementary Information.
