# Supplementary material for: Structural and biochemical analyses of the flagellar expression regulator DegU from Listeria monocytogenes
Source: Sci Rep. 2022 Jul 7;12:10856. doi: 10.1038/s41598-022-14459-5 (PMC9263151; doi:10.1038/s41598-022-14459-5)
Supplement: Supplementary file 1 — Supplementary Information. [file 41598_2022_14459_MOESM1_ESM.pdf]

Supplementary Information

# Structural and biochemical analyses of the flagellar expression regulator DegU from *Listeria monocytogenes*

Han Byeol Oh, Su-jin Lee, Sung-il Yoon[*]

Division of Biomedical Convergence, College of Biomedical Science, Kangwon National University, Chuncheon 24341, Republic of Korea

* Corresponding author

- Sung-il Yoon (E-mail: sungil@kangwon.ac.kr, Phone: +82-33-250-8385, Fax: +82-33-259-5643, Address: 1 Kangwondaehak-gil, Biomedical Science Building A-204, Chuncheon 24341, Republic of Korea)

**Supplementary Table S1.** Crystallographic statistics of the $lmDegU_{DBD}$ structure.

| | $\mathbf{lmDegU_{DBD}}$ |
|---|---|
| **Data collection** | |
| Space group | $P4_32_12$ |
| Cell parameters | a = b = 57.24 Å |
| | c = 145.26 Å |
| Wavelength (Å) | 0.9792 |
| Resolution (Å) | 50.00-2.39 |
| Highest resolution (Å) | 2.43-2.39 |
| No. unique reflections | 10,058 (480) [a] |
| $R_{merge}$ (%) [b] | 7.4 (196.8) [a] |
| $R_{meas}$ (%) [c] | 8.1 (213.4) [a] |
| $R_{pim}$ (%) [d] | 3.0 (80.5) [a] |
| $CC_{1/2}$ [e] | 0.998 (0.605) [a] |
| I/sigma(I) | 29.5 (1.2) [a] |
| Completeness (%) | 98.1 (99.2) [a] |
| Redundancy | 6.9 (6.7) [a] |
| **Refinement** | |
| Resolution (Å) | 50.00-2.39 |
| No. of reflections (work) | 9,526 |
| No. of reflections (test) | 492 |
| $R_{work}$ (%) [f] | 22.7 |
| $R_{free}$ (%) [g] | 25.3 |
| No. atoms | |
| Protein | 1,024 |
| Water | 5 |
| Average B-value ($Å^2$) | 76.6 |
| RMSD bonds (Å) | 0.008 |
| RMSD angles (°) | 0.935 |
| Ramachandran [h] (favored) | 99.3% |
| (outliers) | 0.0% |

[a] Numbers in parenthesis were calculated from data of the highest resolution shell.

[b] $R_{merge} = \Sigma_{hkl}\Sigma_i | I_i(hkl) - \langle I(hkl)\rangle | / \Sigma_{hkl}\Sigma_i I_i(hkl)$

[c] $R_{meas} = \Sigma_{hkl}\{N(hkl)/[N(hkl) - 1]\}^{1/2} \Sigma_i | I_i(hkl) - \langle I(hkl)\rangle | / \Sigma_{hkl}\Sigma_i I_i(hkl)$

[d] $R_{pim} = \Sigma_{hkl}\{1/[N(hkl) - 1]\}^{1/2} \Sigma_i | I_i(hkl) - \langle I(hkl)\rangle | / \Sigma_{hkl}\Sigma_i I_i(hkl)$

[e] Correlation coefficient between intensities from random half-data sets.

[f] $R_{work} = \Sigma| |F_{obs}|-|F_{calc}| | / \Sigma|F_{obs}|$, where $F_{calc}$ and $F_{obs}$ are the calculated and observed structure factor amplitudes, respectively.

[g] $R_{free}$ = as for $R_{work}$, except that 5% of the total reflections were selected at random and omitted from refinement.

[h] Calculated using MolProbity (http://molprobity.biochem.duke.edu).

# Supplementary Fig. S1

```
                   10        20        30        40        50        60        70        80
lmDegU ----MALKIMIVDDHQLFREGIKRILELEDSFEVVAEAENGKNIVAKVREYKPDIVLMDINMPTVNGLDATEMLVRQFPSIKVI
lgDegU ----MTLKIMIVDDHQLFREGLKHILETEDSFEVVLEAEDGKNIVSKVREASPDVILMDINMPSVNGLDATEMLIRQFPSAKII
ooDegU MDAQKEVNIVLIDDHKLFREGVKRILEFEPAFKVVAEADDGTDAANLVRDNNPDVVLMDINMPKINGVQATADLLRQFPHTKII
bsDegU ---MTKVNIVIIDDHQLFREGVKRILDFEPTFEVVAEGDDGDEAARIVEHYHPDVVIMDINMPNVNGVEATKQLVELYPESKVI
gsDegU ----MKTRIAIIDDHSLFREGIKRILEFEGDFEVVAEGSDGSEVLSIVETYRPDLVLMDINMPDMNGVEATKQLIETYPETKVV

               90       100       110       120       130       140       150
lmDegU VLTIHDTDEYVTEALRAGAVGYLLKEMDAHELVEAVKIVDNGGAYIHPRVAIKLIREYRHLASTNTSQGVYGYQQPEV
lgDegU MLSIHDSDEFVSSALKTGAVGYLLKEMDANELIEAIKVVYKGGAYIHPKAAIKLIREYRNLASSSSAQGNYGYRQPEV
ooDegU ILSIHDDENYVTHALKTGAQGYLLKEMDAEALVEAIKVVSEGGSYLHPRITHNLVKEYRRLARENTSSLANR--HVEH
bsDegU ILSIHDDENYVTHALKTGARGYLLKEMDADTLIEAVKVVAEGGSYLHPKVTHNLVNEFRRLATSGVSAHPQHEVYPEI
gsDegU VLSIHDDEHYVMRALQTGATGYLLKEMDADTLIEAVKVVAEGGSYLHPKVTHNLIREYRRLTSEKG----GVAVKQEV
```

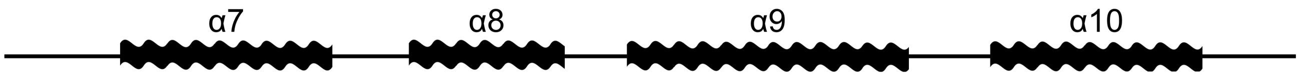


```
       160       170       180       190       200       210       220
lmDegU  KMPLHILTHRECEVLQLLTDGKSNRGIGETLFISEKTVKNHVSSILQKMKVNDRTQAVVTAIKHGWVYIR
lgDegU  KMPLHLLTHRECEVLQLLTDGKSNRSIGETLFISEKTVKNHVSSILQKMKVNDRTQAVVTAIKHGWVYIR
ooDegU  RKPLHLLTRRECQVLQLLAEGKSNRAVAESLYISEKTVKNHVSNILQKMNVNDRTQAVVCAIRKGWVEV-
bsDegU  RRPLHILTRRECEVLQMLADGKSNRGIGESLFISEKTVKNHVSNILQKMNVNDRTQAVVVAIKNGWVEMR
gsDegU  RRPLHLLTRRECEVLQLLADGKSNRAIGEALYISEKTVKNHVSSILQKLNVNDRTQAVVVAIKNGWVEVR
```

**Supplementary Figure S1. Sequence alignment of lmDegU and its orthologs [*Listeria grayi* DegU (lgDegU), *Oceanobacillus oncorhynchi* DegU (ooDegU), *Bacillus subtilis* DegU (bsDegU), *Geobacillus stearothermophilus* DegU (gsDegU)].** The putative dsDNA-binding residues of lmDegU (R168, K194, K197, N198, and R212) that were described in the text are indicated by magenta circles. The phosphorylation site (D55) and dimerization interface residues of lmDegU are colored red and blue, respectively. The secondary structures of $lmDegU_{DBD}$ are represented by waves (α-helices) and lines (coils) above the lmDegU sequence.

# Supplementary Fig. S2

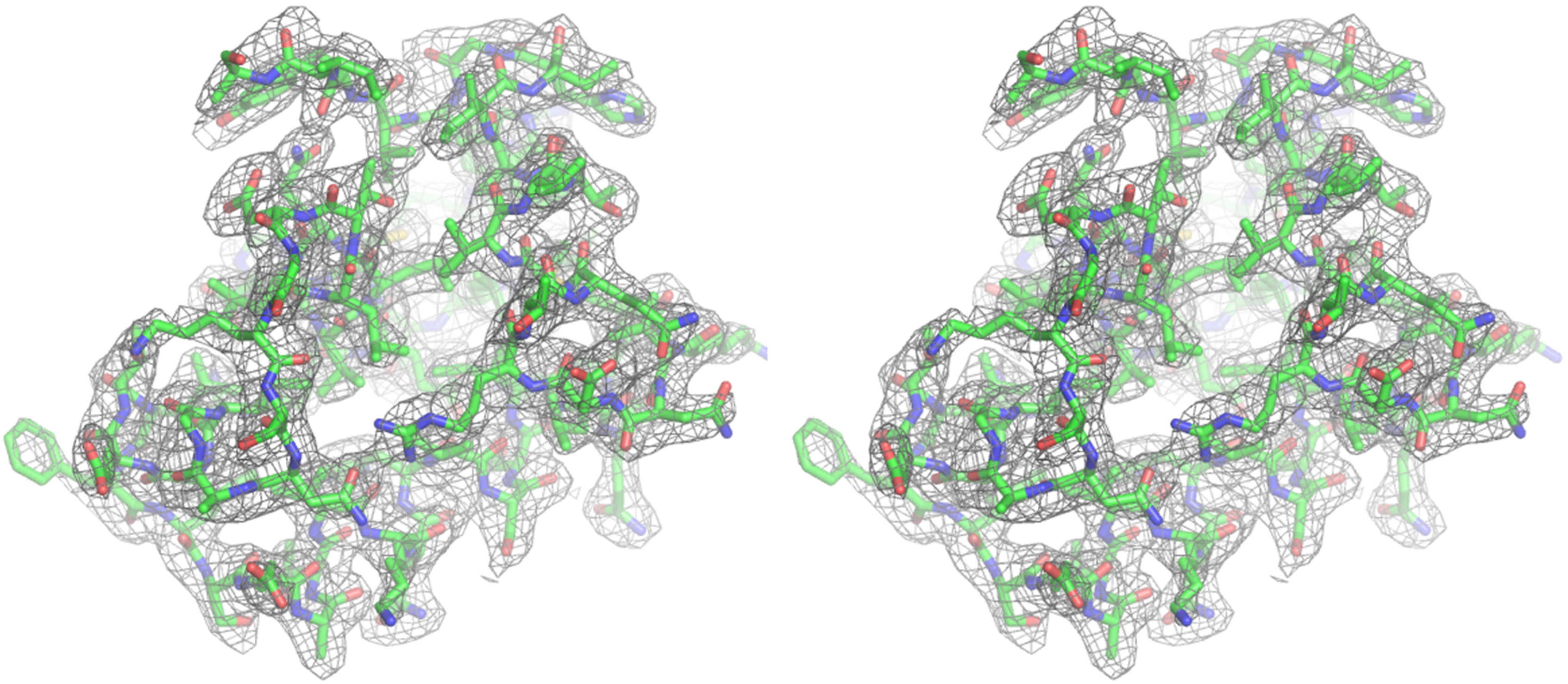

**Supplementary Figure S2. Stereoview of the electron density map (gray wires; 1σ in 2Fo−Fc map) for the lmDegU residues of chain A (green sticks) in the orientation showing the dimerization interface.** The orientation of lmDegU chain A in this figure is identical to that in Fig. 2B.

# Supplementary Fig. S3

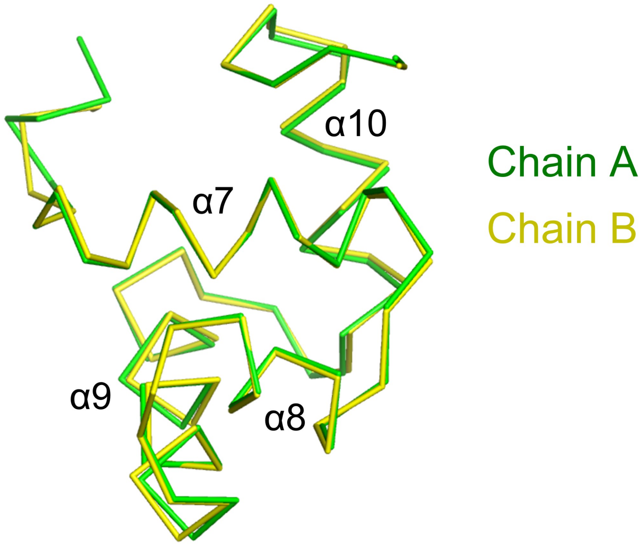


**Supplementary Figure S3. Structural overlay of lmDegU$_{DBD}$ chains A and B (green and yellow Cα traces, respectively) in the asymmetric unit of the lmDegU$_{DBD}$ crystal.**

# Supplementary Fig. S4

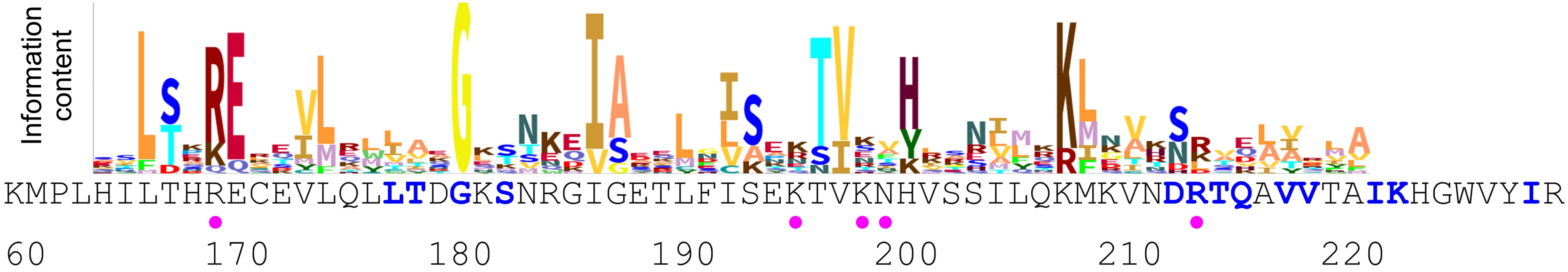


**Supplementary Figure S4. Sequence logo of GerE (PF00196) family members.** The sequence logo was generated in the Skylign server (https://skylign.org/) using 26 GerE family sequences obtained from the Pfam database (http://pfam.xfam.org/family/PF00196). The lmDegU$_{DBD}$ sequence is shown under the sequence logo. The putative dsDNA-binding residues of lmDegU (R168, K194, K197, N198, and R212) that were described in the text are indicated by magenta circles. The dimerization interface residues of lmDegU are colored blue.

# Supplementary Fig. S5

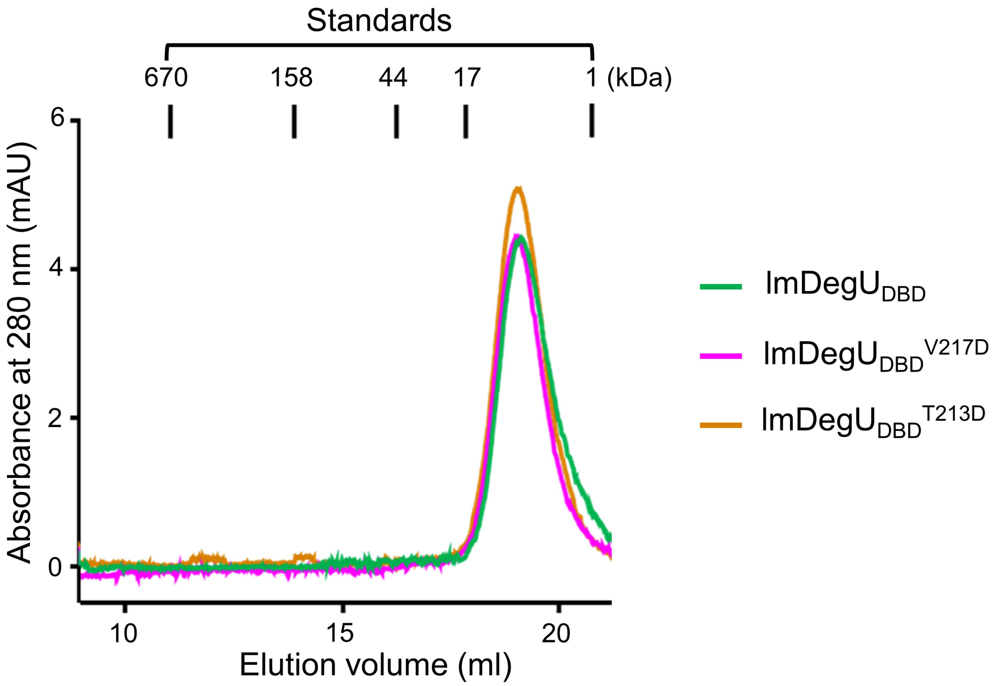


**Supplementary Figure S5. Gel-filtration chromatography analysis of lmDegU$_{DBD}$, lmDegU$_{DBD}^{V217D}$, and lmDegU$_{DBD}^{T213D}$.** In gel-filtration chromatography, lmDegU$_{DBD}$ (calculated molecular weight, 8.6 kDa) was eluted as a monomer. The gel-filtration profiles of lmDegU$_{DBD}^{V217D}$ and lmDegU$_{DBD}^{T213D}$ were similar to that of lmDegU$_{DBD}$, suggesting that the V217D and T213D mutations do not significantly modulate the folding of the lmDegU$_{DBD}$ protein. The gel-filtration profile in the figure is representative of three independent experiments that yielded similar results.

# Supplementary Fig. S6

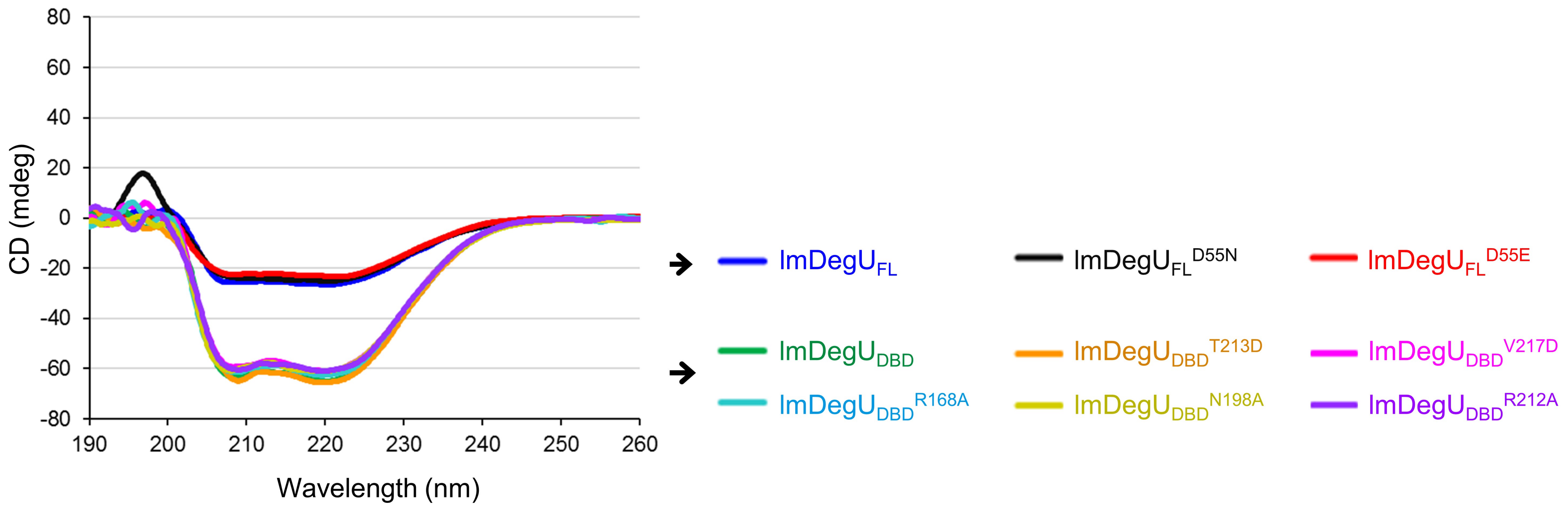


**Supplementary Figure S6. CD spectra for lmDegU$_{FL}$, lmDegU$_{DBD}$, and their mutants.** To confirm that mutation does not induce lmDegU protein unfolding, far-UV CD spectra were obtained at 25°C for lmDegU variants (0.5 mg/ml).

# Supplementary Fig. S7

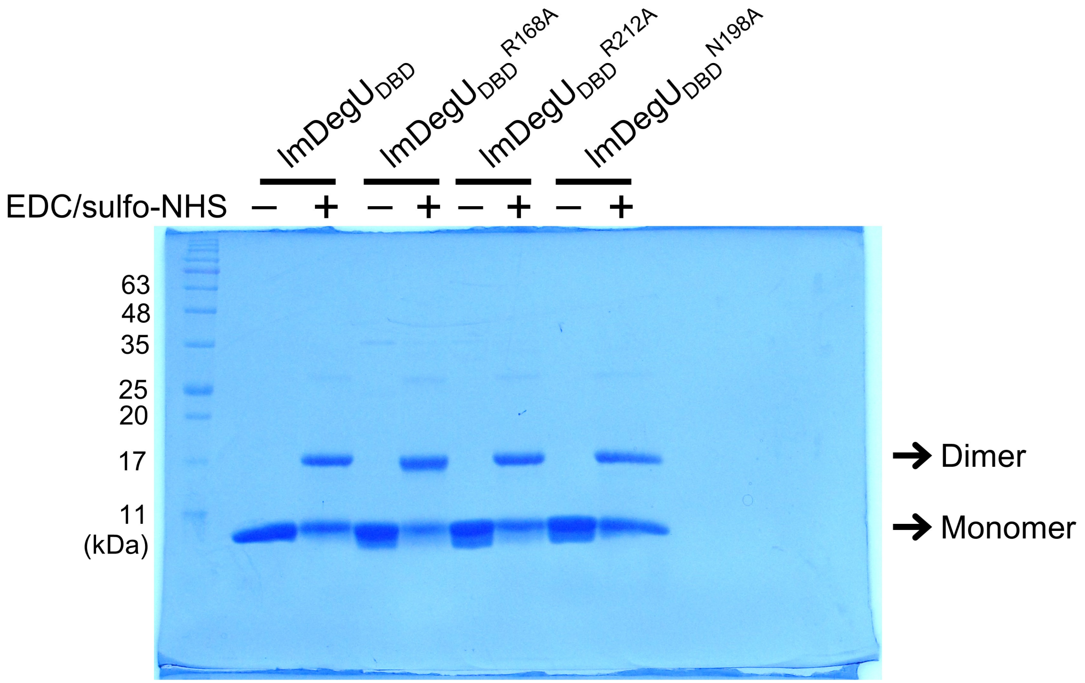


**Supplementary Figure S7. Similar dimerization levels of lmDegU$_{DBD}$ and DNA binding-deficient mutants (lmDegU$_{DBD}^{R168A}$, lmDegU$_{DBD}^{R212A}$, and lmDegU$_{DBD}^{N198A}$) in chemical crosslinking experiments.** The R168 and N198 residues are located outside the dimerization interface of lmDegU$_{DBD}$. The R212 residue is located at the periphery of the dimerization interface. lmDegU$_{DBD}$ or its mutant was crosslinked using EDC and sulfo-NHS and analyzed by SDS-PAGE. Protein bands were identified by Coomassie brilliant blue staining. The gel image is representative of three independent experiments that yielded similar results.

# Supplementary Fig. S8

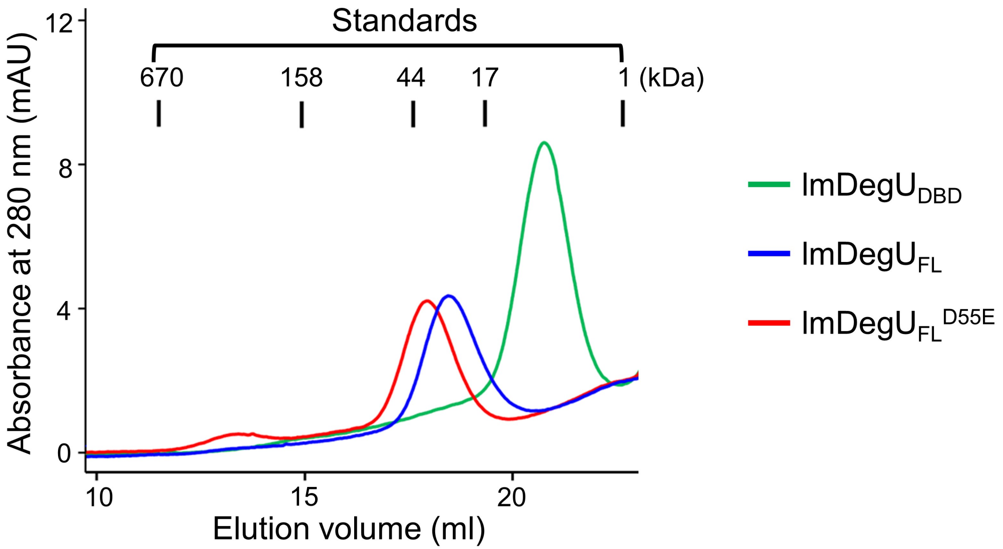


**Supplementary Figure S8. Gel-filtration chromatography analysis of lmDegU$_{DBD}$, lmDegU$_{FL}$, and lmDegU$_{FL}^{D55E}$.** In gel-filtration chromatography, lmDegU$_{DBD}$ (calculated molecular weight, 8.6 kDa) and lmDegU$_{FL}$ (calculated molecular weight, 26.9 kDa) were eluted as monomers, suggesting that lmDegU$_{DBD}$ and lmDegU$_{FL}$ homodimerize with low affinities. The lmDegU$_{FL}^{D55E}$ phosphomimetic was eluted earlier than lmDegU$_{FL}$. The gel-filtration profile is representative of three independent experiments that yielded similar results.

# Supplementary Fig. S9

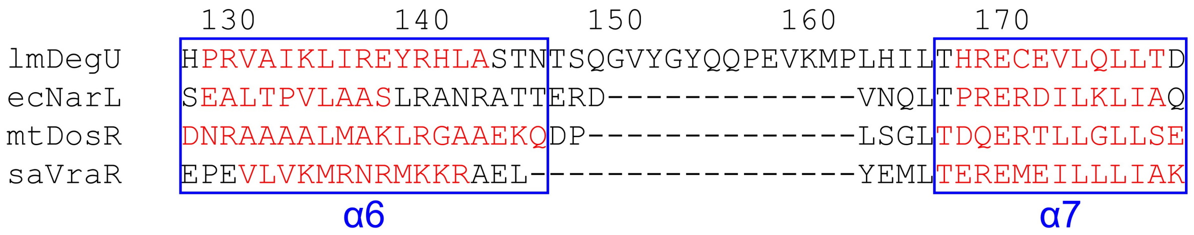


**Supplementary Figure S9. Extended interdomain region of lmDegU.** The sequences of lmDegU and its homologs (*Escherichia coli* NarL, ecNarL; *Mycobacterium tuberculosis* DosR, mtDosR; *Staphylococcus aureus* VraR, saVraR) are aligned at the α6 and α7 helices and the α6-α7 loop in or near the interdomain region. The sequences corresponding to the α6 and α7 helices are colored red in blue boxes. The lmDegU sequences corresponding to the α6 and α7 helices were identified by secondary structure prediction (http://raptorx.uchicago.edu/StructurePropertyPred/predict/) and $lmDegU_{DBD}$ structure analysis, respectively. The α6 and α7 helices of ecNarL, mtDosR, and saVraR were assigned based on their crystal structures (PDB ID 1RNL, 3C3W, and 4GVP, respectively).

# Supplementary Fig. S10

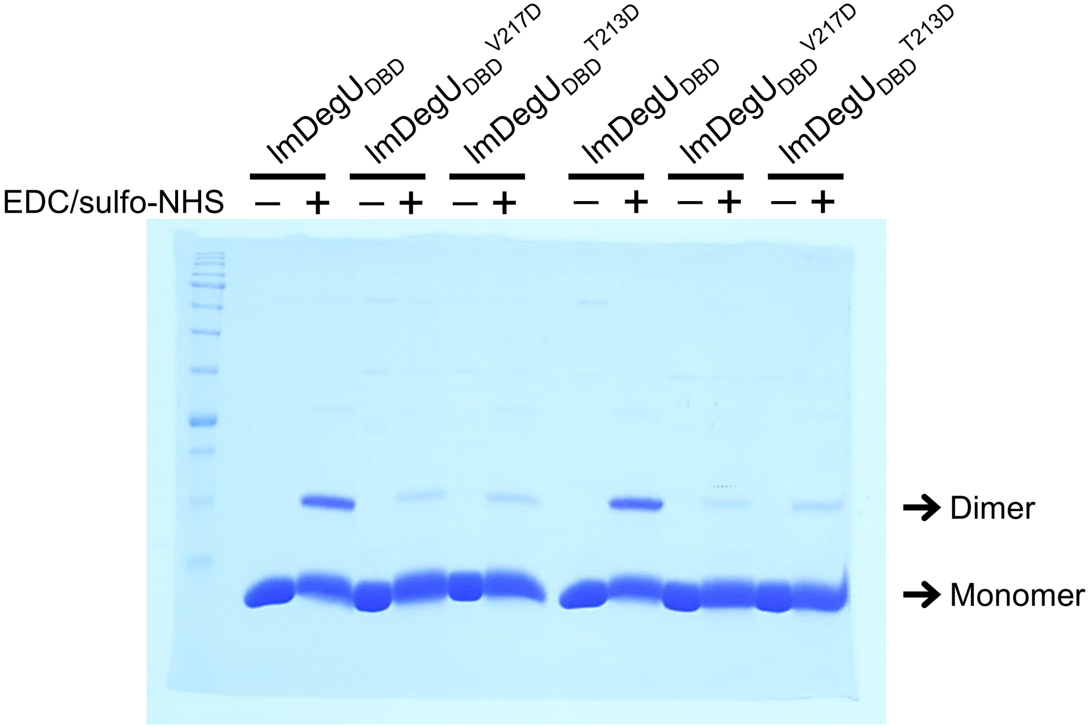


**Supplementary Figure S10. Full-length gel of Fig. 2C.** Identical crosslinking reactions were analyzed by SDS-PAGE in duplicate side-by-side.

# Supplementary Fig. S11

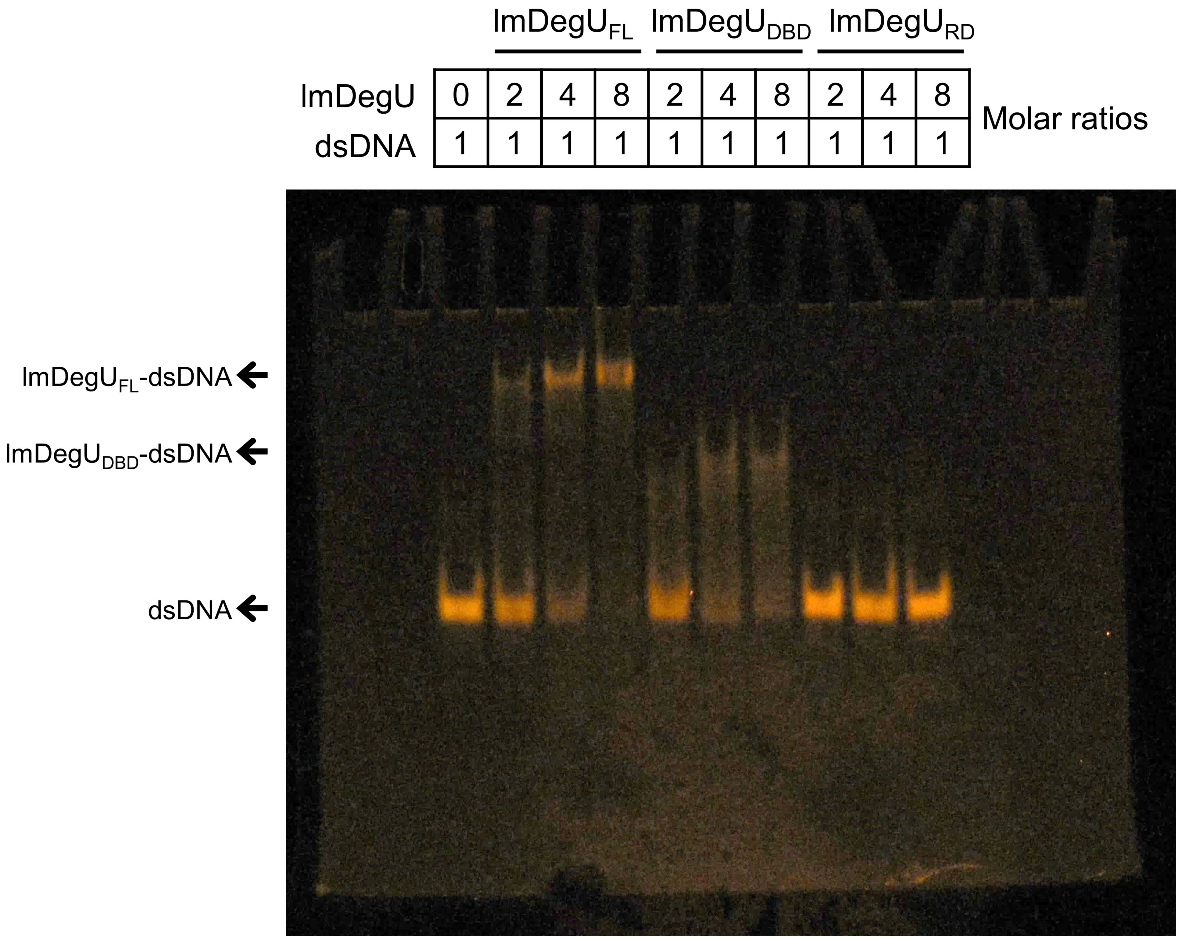


**Supplementary Figure S11. Full-length gel of Fig. 5B.**
